# Comprehensive Analysis of MYB Gene Family and Their Expressions Under Abiotic Stresses and Hormone Treatments in *Tamarix hispida*

**DOI:** 10.3389/fpls.2018.01303

**Published:** 2018-09-19

**Authors:** Tengqian Zhang, Yulin Zhao, Yucheng Wang, Zhongyuan Liu, Caiqiu Gao

**Affiliations:** ^1^State Key Laboratory of Tree Genetics and Breeding, Northeast Forestry University, Harbin, China; ^2^Taiyuan Botanical Garden, Taiyuan, China

**Keywords:** abiotic stress, MYB transcription factor, gene expression, *Tamarix hispida*, Na^+^ and K^+^ content

## Abstract

The MYB transcription factors (TFs) is a plant TF families, which involves in hormone signal transduction, and abiotic stress tolerance, etc. However, there are few studies on the MYB TFs family and its regulatory mechanism in *Tamarix hispida*. In this study, 14 *MYB* genes (named *ThMYB1 - ThMYB14*) were cloned and characterized from *T. hispida*. The transcription profiles of *ThMYBs* in *T. hispida* under different abiotic stress conditions were monitored using qRT-PCR. Most of studied *ThMYBs* were significantly downregulated and/or upregulated by salt and osmotic stress, ABA, GA3 and JA treatments in at least one organ. Especially, *ThMYB13* was induced in the leaves and roots of *T. hispida* when exposed to NaCl treatment at all study periods, indicating that it may involve in salt stress. To further study *ThMYB1*3 function, *ThMYB1*3 overexpression and knock-down plants and control plants transformed with an empty pROKII were obtained using a transient transformation system. Overexpression of *ThMYB1*3 in *T. hispida* displayed the lowest O^2-^, H_2_O_2_ and MDA accumulation, minimal cell death, the most stable K^+^/Na^+^ ratio and the lowest electrolyte leakage rate among the three kinds of transient expression in *T. hispida*. Conversely, the RNAi-silencing, transiently transformed plants displayed the opposite physiological changes. Therefore, *ThMYB1*3 might play a role in salt stress tolerance in transgenic *T. hispida* plants.

## Introduction

The MYB TFs is most abundant in plants (Peng et al., 2016), which contains the MYB domain serving as DNA binding. MYB TFs is classified according to the repeats present that varying from 1 to 4 in their sequences. Therefore, the MYB is divided with 4 groups, 1R-, R2R3-, 3R- and 4RMYB (Roy, 2016).

In previous studies, the *MYB* family genes, *R2R3 MYBs*, were involved in diverse processes, including cell cycle control, hormone signaling, secondary metabolism, meristem formation and cellular morphogenesis. Additionally, some *MYB* genes were found to regulate responses to abiotic stress (Cao et al., 2013). For, *AtMYB96* induced pathogen resistance through the pathway of ABA signaling that regulating stomatal movement, increased tolerance to drought and disease (Seo et al., 2009; Seo and Park, 2010). Moreover, Arabidopsis *AtMYB15* and *AtMYB44*, were found to involve in stomatal closure to improve drought tolerance (Jung et al., 2008; Ding et al., 2009). The silencing of *GbMYB5* reduced antioxidant enzyme activities and proline content, leading to decreased the drought tolerance. Furthermore, overexpression of *GbMYB5* in tobacco improved tolerance to drought accompanied with decreased water loss, elevated the proline level and ROS scanvenging activities; meanwhile, the expression of *SOD, CAT, GST, SAMDC* and *ADC1* were significantly induced (Chen et al., 2015).

The *MYB* gene had been found to involve in plant salt stress responses. The plants overexpression of *AtMYB20* increased salt tolerance, however, the plants repressing *AtMYB20*-SRDX showed decreased tolerance to salt stress (Cui et al., 2013). *AtMYB73* could be induced by salt stress. The peak expression of *AtMYB73* occurred at NaCl treatment for 6 h. In addition, *AtMYB73* played a negative role in SOS induction in Arabidopsis (Kim et al., 2013). Arabidopsis plants expressing *TaMYB3R1* produced more rosette leaves during the stage of vegetative growth; whereas they produced more inflorescences at the reproductive stage. The transformed lines showed improved tolerance to salt and drought treatments (Cai et al., 2015).

Additionally, some MYB TFs also participate in light, low-temperature, and osmotic stress induction responses. *AtMYB18/LAF1* and *AtMYB38* control hypocotyl elongation responding to far-red light (Yang et al., 2009) and blue (Hong et al., 2008) in seedlings, respectively. The *OsMYB4* gene from rice enhanced frost tolerance and improved germination in transgenic barley plants under unfavorable conditions (Soltész et al., 2012). Arabidopsis expressing the apple *MdMYB10* gene displayed enhanced tolerance to osmotic stress (Gao et al., 2011).

*Tamarix hispida* is a woody halophyte. This kind of perennial shrub or small tree is highly resistant to drought and soil salinity. This plant can absorb a high amount of salt from the soil and can accumulate it in the cells, with the transfer of salt achieved upon harvest. This resistance of *T. hispida* makes it an ideal material for cloning salt-tolerant genes. Therefore, in order to screen for genes with excellent resistance to abiotic tolerance, 14 *T. hispida MYBs* were cloned and their expression under salt or osmotic stress was analyzed by qRT-PCR. Further, *ThMYB13* was selected for transiently transformed into *T. hispida*. The results showed that *ThMYB13* could significantly improve salt tolerance of transgenic *T. hispida*, and it was an excellent salt tolerance gene. This investigate will provide new insights into the function of *ThMYBs* in tolerance to salt.

## Materials and Methods

### Plant Materials and Growth Conditions

*Tamarix hispida* seeds were collected from Turpan Desert Botanical Garden of Chinese Academy Sciences (Xinjiang province, China) and were planted in a mixture of sand and turf peat (1:2, v/v) with the conditions of 14 h light/10 h darkness photocycle and 70–75% relative humidity under the temperature of 24°C. The seedlings (approximately 5–6 cm in height, 2-month old) with good growth and uniform size were used for stress treatment. The seedlings were irrigated every 24 h on roots with the solution of 20% (w/v) PEG_6000_, 0.4 M NaCl, 100 μM ABA, 50 μM GA3 or 100 μM JA solution and were harvested at 6, 12, 24, 48, and 72 h. Meanwhile, the *T. hispida* plants irrigated with fresh water were served as the controls. After these treatments, roots or leaves from about 20 seedlings were pooled together at each time point, and stored at -80°C.

### Identification of the MYB Genes in *T. hispida*

Seven transcriptome libraries had been constructed with *T. hispida* plants with 2-month-old, including 4 root transcriptomes treated respectively with NaHCO_3_ for 0, 12, 24, and 48 h, and 3 leaf transcriptomes treated with NaHCO_3_ for 0, 12, and 24 h (Wang et al., 2014). The unigenes were analyzed using BLASTX program for searching the Swiss-Prot and NR databases. The phrase “MYB transcription factor (TFs)” was searched against the unigenes with functional annotation to identify *MYB* family genes. Then, the *ThMYB* gene*s* with complete open reading frames (ORFs) were selected by ORF finder^1^. The Mol.Wt and theoretical pI of the proteins encoded by *ThMYBs* were studied by ProtParam^2^.

### Phylogenetic and Sequence Conservation Analysis

Arabidopsis MYB proteins were retrieved from TAIR database^3^. The MEGA5.0 software (Tamura et al., 2011) was used to systematically analyze the MYB proteins of Arabidopsis and *T. hispida*. Neighbour-joining (NJ) was employed to predict the phylogenetic tree, which was subjected to a Bootstrap method with 1000 replications (Tamura et al., 2011). Multiple sequence alignments was performed by Clustal X to search the conserved regions of the ThMYBs with the gap extension penalties and a gap open of 10 and 0.1, respectively (Thompson et al., 1997). ExPASy-PROSITE^4^ was used to predict the conserved domains of ThMYBs protein sequences.

### RNA Extraction and qRT-PCR Analysis

Total RNA was extracted from *T. hispida* samples using an RNA extraction kit (BioTeke Corporation, Beijing, China). About 1 μg of total RNA was transcribed into cDNA. The synthesized cDNA was diluted to 10-fold with sterile water to act as the template for qRT-PCR. In addition, actin (FJ618517), *α-tubulin* (FJ618518), and β-tubulin (FJ618519) were used as the internal reference genes in *T. hispida* to normalize the templates in the PCR reactions. All primer sequences used were shown as **Supplementary Table S1**. qRT-PCR was conducted on an Opticon 2 system (Bio-Rad). The PCR reaction with a volume of 20 μl contains the followings: 2 μl of template cDNA, 0.5 μM of each reverse and forward primer, 10 μl of SYBR Green Real-time PCR Master Mix (Toyobo). PCR program was set as following conditions: 95°C for 30 s, followed by 45 cycles of 95°C for 15 s, 58°C for 15 s, 72°C for 30 s and 1 s at 80°C for reading plate. All the reactions were performed in triplicate. Expression levels were determined according to the 2^-ΔΔCt^ (Livak and Schmittgen, 2001).

### Vector Construction and Transient Expression of *ThMYB13* in *T. hispida*

To verify the salt tolerance function of *ThMYB* genes, the overexpression and RNAi vectors of *ThMYB13* were constructed. The CDS of *ThMYB13* was inserted into pROKII driving by 35S CaMV promoter to overexpress *ThMYB13* (35S:MYB). An inverted repeat truncated CDS of *ThMYB13* with the length of 246 bp was inserted into pFGC5941 (Kerschen et al., 2004) at the two sides of the CHSA intron (pFGC:MYB) for silencing *ThMYB13* expression. All primer used are shown in **Supplementary Table S2**. The genetic transformation of *T. hispida* plants was carried out following Ji et al. (2014). In brief, 40 day tissue cultures of *T. hispida* seedlings with similar sizes were used for genetic transformation. The seedlings were incubated in a solution for transformation [1/2 MS + 150 μM acetosyringone + 3% (w/v) sucrose + 0.01% (w/v) Tween 20 + 0.6 OD_600_ *Agrobacterium tumefaciens*, pH 5.6] shaking with 120 rpm at 25°C for 6 h. The seedlings were washed twice using distilled water and planted vertically on 1/2 MS solid medium. In total, 3 types of transiently transformed plantlets were cultured, including plants transiently transformed with 35S:MYB to overexpress *ThMYB13* (OE), with pFGC:MYB for silencing *ThMYB13* (RNAi) expression, or control (Con) plants transformed with the empty pROKII plasmid.

### Biochemical Staining and Physiological Measurement of Transformed Plants

After culturing for 36 h on 1/2 MS agar medium, the seedlings from transient transgenic *T. hispida* (OE, Con and RNAi) were transferred to new 1/2 MS medium (as control) or 1/2 MS with 150 mM NaCl stress 2 h for biochemical staining, and then these seedlings were incubated with NBT, DAB or Evans blue solutions. The procedures for NBT and DAB staining were according to Zhang et al. (2011). Evans blue staining was performed as described by Yang et al. (2014). H_2_O_2_ was measured following the protocol of Dal Santo et al. (2012).

In addition, after culturing for 36 h on 1/2 MS solid medium, the plants were transferred to 1/2 MS medium with 150 mM NaCl for stress for 12, 24, or 36 h. The plants were harvested for qRT-PCR and physiological analyses. The expression of *ThMYB13* in the transgenic plants were measured with qRT-PCR as described above. MDA measurements were conducted descried by Wang et al. (2010), while the electrolyte leakage measurement was conducted as described by Ji et al. (2014). The determination of the Na^+^ and K^+^ content was described in Chen et al. (2005) and Ma et al. (2011). All experiments were conducted with triplicate.

### Statistical Analysis

The data were analyzed with the Statistical Software Package for Social Science (SPSS) version 17.0. Using Student’s *t*-test to compare the data, if *P* < 0.05, the data were considered significantly different. ^∗^Indicates significant difference (^∗^*P* < 0.05).

## Results

### Identification the *ThMYB* Genes in *T. hispida*

A total of 238 unigenes were found to be the MYB family genes from the seven transcriptomes. BLASTX analysis was performed, and 14 unigenes of the *ThMYBs* with full ORF were finally obtained and searched in the NR protein database. In addition, these 14 *ThMYB* genes were named *ThMYB1 to ThMYB14*. The proteins of these 14 *ThMYBs* ranged from 284 to 554 amino acids residues, their pI were between 5.05 and 9. 66 and the Mol.Wt ranging from 30.6 to 61.8 kDa (**Table 1**).

**Table 1:** *MYB* genes in *T. hispida*.

Gene symbol	Length of gene coding region (bp)	Deduced protein amino acid residues (aa)	pI	Mol.Wt (kDa)
*ThMYB1*	873	290	8.81	32.7
*ThMYB2*	1092	363	6.16	41.2
*ThMYB3*	1191	396	5.39	43.4
*ThMYB4*	1209	402	6.98	45.2
*ThMYB5*	1095	364	8.27	39.2
*ThMYB6*	855	284	9.66	30.6
*ThMYB7*	936	311	9.05	35.1
*ThMYB8*	1014	337	5.05	37.5
*ThMYB9*	1230	409	5.61	45.7
*ThMYB10*	897	298	6.2	31.9
*ThMYB11*	930	309	8.37	35.0
*ThMYB12*	1665	554	6.29	61.8
*ThMYB13*	1014	336	8.23	36.6
*ThMYB14*	900	299	5.14	33.0


*Including gene symbol, length of gene coding region (bp), deduced protein amino acid residues (aa), isoelectric point (pI), and molecular weight.*

### Sequence Conservation Analysis of *ThMYBs*

The conserved domains of 14 ThMYB protein sequences were analyzed using ExPASy-PROSITE. The results showed that the structural domain of each ThMYB protein had at least one MYB conserved domain. To further analyze the similarities and characterization of the protein sequences of 14 ThMYB, their deduced amino acid sequences of were sequenced by Clustal X software. As shown in **Supplementary Figure S1**, 14 ThMYB protein sequences have a conserved MYB domain, and the conserved domains have high similarity. The N-terminus of *ThMYB2, 3, 4, 8, 9*, and *13* protein sequences contain two conserved MYB domains. The conserved domain of *ThMYB12* exists at the end of the peptide chain. The conserved domains of *ThMYB5, 6, 7, 10*, and *11* were located in the middle of the peptide chain. *ThMYB1* and *ThMYB14* contain a conserved domain of MYB-type in the N-terminus.

Further analysis of the sequences of these conserved domains revealed that *ThMYB2, 3, 4, 8, 9*, and *13* all contained the R2R3 conserved domain (**Supplementary Figure S2**). It was suggested that these 6 *ThMYB* genes belong to R2R3-MYB family, in which the first tryptophan in the R3 domain of *ThMYB8* is replaced by leucine (L) and the first tryptophan in the R3 domain of the other five proteins is replaced by phenylalanine (F). In addition, the peptide chains of *ThMYB5, 6, 7, 10*, and *11* contain a highly conserved domain of MYB-type HTH DNA in the middle (**Supplementary Figure S3**).

### Phylogenetic Analysis of *ThMYBs*

**FIGURE 1:**
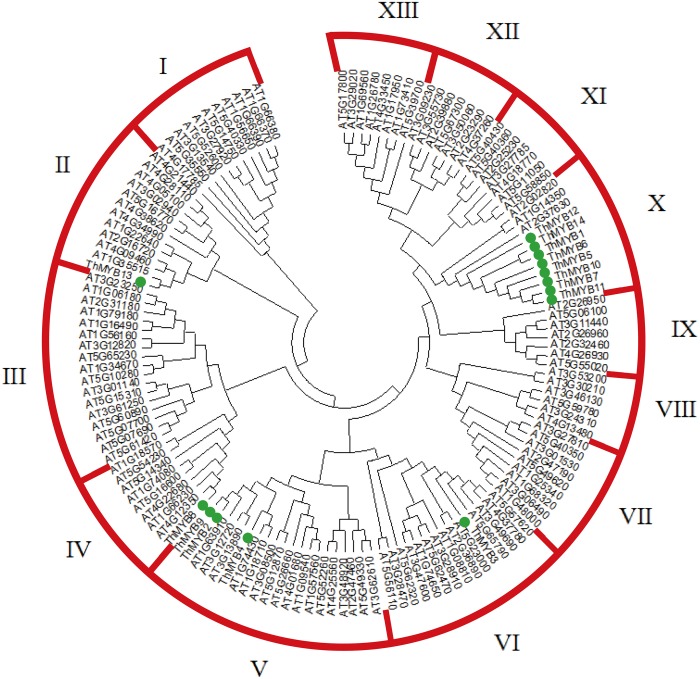
The phylogenetic tree of MYB proteins from *T. hispida* and Arabidopsis. In total, 14 MYB proteins in *T. hispida* and 125 in Arabidopsis were analyzed using Clustal X 2.0, and the neighbor-joining tree was constructed using MEGA 5.0. The bootstrap value was 1000 replicates.

A NJ phylogenetic tree of 14 *T. hispida* ThMYB proteins and 125 Arabidopsis MYB proteins were constructed (**Figure 1**). The phylogenetic distribution indicated that MYB proteins can be classified into 13 groups, while the 14 ThMYB TFs were divided into 5 subgroups: class II, IV, V, VI, and X. Among them, class X was the largest subgroup and contained 8 ThMYB proteins, ThMYB1, 5, 6, 7, 10, 11, 12, and 14. All of these proteins contained only one conserved domain. The other ThMYB proteins (including ThMYB 2, 3, 4, 8, 9, and 13) that contained two conserved MYB domains were clustered into four individual clades.

### Relative Expression Levels of *ThMYB* Genes in Roots and Leaves of *T. hispida*

**FIGURE 2:**
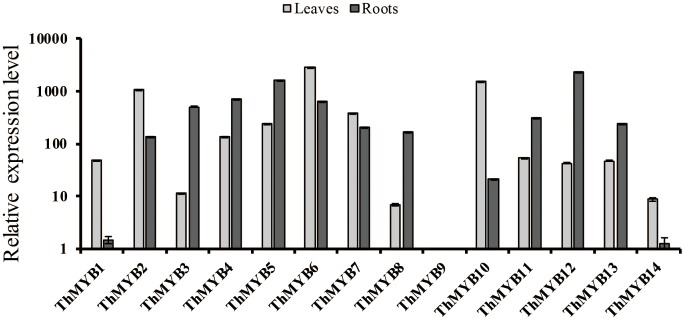
Relative expression levels of the 14 *ThMYBs* in leaf and root tissues of *T. hispida* under normal conditions. The *ThMYB9* gene, which had the lowest expression levels (the highest delta Ct value) in both of the tissues of leaf and root, were assigned as 1. The expression levels of other *ThMYB* genes were plotted relative to the expression of *ThMYB9*. The y-axis shows the relative expression level of *ThMYB* genes, and the x-axis shows the *ThMYB* genes. The relative expression levels of different *ThMYBs* were log10-transformed.

To study the expression patterns of these 14 *ThMYB* genes in *T. hispida* leaves and roots without stress, their expressions were measured by qRT-PCR. The lowest transcript level genes (i.e., the highest delta Ct value) were set as 1 to normalize the transcript levels of the other *ThMYBs* (**Figure 2**). The relative expression levels of the 14 *ThMYBs* exhibited marked differences in leaves and roots. Interestingly, *ThMYB9* was the least abundant in both the leaves and roots. However, *ThMYB6* was the most abundant gene in leaves, and *ThMYB12* was the most abundant gene in roots. The abundance of *ThMYB6* was 2939 times higher than the lowest abundance (*ThMYB9*) in leaves, and the abundance of *ThMYB12* was 2391 times greater than *ThMYB9* in the roots, indicating that *ThMYB6* in the leaves and *ThMYB12* in the roots might play important functions than the other *ThMYBs*.

### Expression Analysis of *ThMYBs* Under Different Abiotic Stresses and Hormone Stresses

To analyze the stress response function of *ThMYBs*, the expression profiles of 14 *ThMYBs* under different stress, including high salt, osmotic stress, and hormones treatments (ABA, GA3, JA) were analyzed by qRT-PCR.

### Abiotic Stresses

**FIGURE 3:**
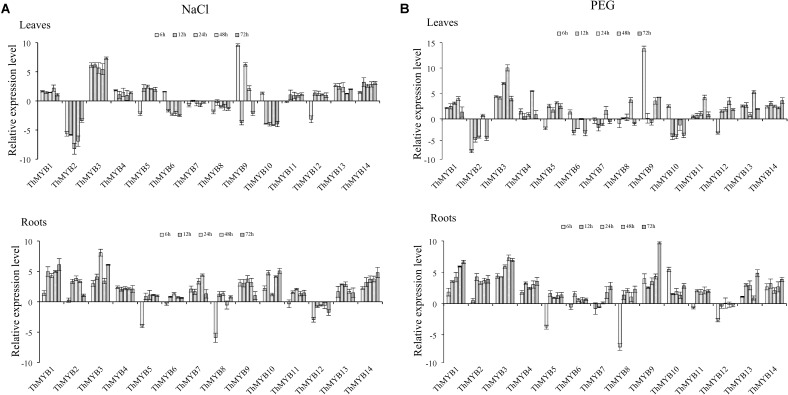
Expression analysis of the 14 *ThMYBs* responding to abiotic stresses (NaCl, PEG) in leaves and roots. **(A)** NaCl. **(B)** PEG. The relative expression level = transcription level under stress treatment/transcription level under control conditions. All relative transcription levels were log2-transformed. The error bars were obtained from multiple replicates of qRT-PCR.

Under NaCl stress, most of *ThMYB* genes had upregulated expression in the leaves. Especially, *ThMYB1, 3, 4, 11, 13* and *14* had all induced expression at all stress points. The expression of *ThMYB9* was significantly upregulated at most stress times (6, 24, and 48 h). The relative expression levels of *ThMYB5* and *ThMYB12* in leaves were upregulated at 12–72 h but downregulated rapidly at 6 h after salt stress. In contrast, *ThMYB6* and *ThMYB10* were induced at 6 h and inhibited with the other studied stress times. In the roots, the expression patterns of *ThMYB1, 3, 4, 11, 13*, and *14* were the same as those in leaves, and they were upregulated at all stress points. However, *ThMYB*6, *10* and *ThMYB12* were significantly different from those in the leaves. The expressions of *ThMYB6* and *10* were mainly upregulated at all stress points, and *ThMYB12* was downregulated at all studied time points. In addition, the transcript of *ThMYB8* was significantly downregulated at 6 h, and the lowest expression was only 1.65% of the control (**Figure 3A**).

Under PEG stress, the expressions of *ThMYB1, 3, 13*, and *14* were significantly upregulated at all stress points in the leaves. In addition, the most induced gene was *ThMYB9*, in which the peak expression level was 14,563-fold (6 h) that of the control. The relative expressions of *ThMYB5* and *ThMYB12* in leaves were also upregulated after stress at 12 h. In contrast, some *ThMYB* genes showed downregulated expression after PEG stress. Especially, the expression of *ThMYB2* was significantly downregulated at most stress times (except 48 h), which was only 1.24% of the control at PEG stress at 6 h. Similarly, *ThMYB6* and *ThMYB10* were downregulated with most of the studied stress times (except 6 h). In the roots, almost all of the genes were significantly upregulated at all stress points, except for *ThMYB5, 7, 8, and 12*. Especially, with the prolonged stress time, the relative expression level of *ThMYB1* gradually increased. The expressions of *ThMYB5, 8*, and *12* were significantly downregulated at 6 h. However, at 12–72 h, the relative expressions of *ThMYB5* and *8* were upregulated, while the expression of *ThMYB12* did not change significantly (**Figure 3B**).

### Hormone Treatments

**FIGURE 4:**
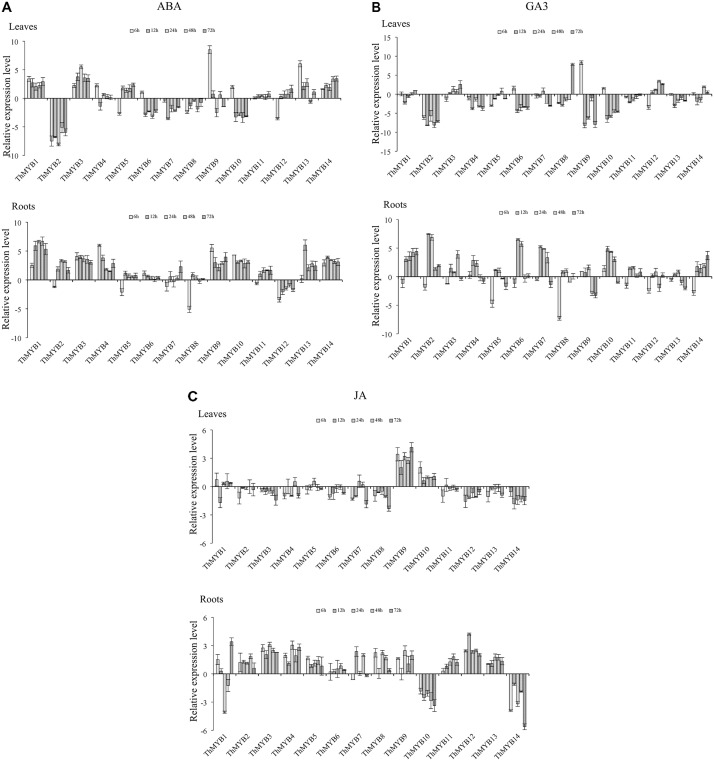
Expression analysis of the 14 *ThMYBs* responding to hormones treatments (ABA, GA3, JA) in leaves and roots. **(A)** ABA. **(B)** GA3. **(C)** JA. The relative expression level = transcription level under stress treatment/transcription level under control conditions. All relative transcription levels were log2-transformed. The error bars were obtained from multiple replicates of the qRT-PCR.

Under ABA treatment, the transcripts of *ThMYB11* were not significantly differentially regulated at all stress points in the leaves. However, most the *ThMYB* genes expressed were significantly different after ABA stress. Especially, *ThMYB1, 3, and 14* showed significantly upregulated expression at all stress points, while *ThMYB2, 7, and 8* were downregulated at all studied stress times. The relative expression of *ThMYB5* and *ThMYB12* in leaves was upregulated at 12-72 h but downregulated rapidly at 6 h after ABA stress. In contrast, *ThMYB6* and *ThMYB10* were induced at 6 h and inhibited with the other studied stress times. In the roots, the transcripts of all *ThMYB* were classified into three groups. One group was included by *ThMYB1, 3, 4, 6, 9, 10, 13*, and *14* and was clearly upregulated at all stress points. The second *ThMYB* group composed of *ThMYB2, 5, 7, 8*, and *11* was upregulated at 12–72 h but downregulated at 6 h after treatment of ABA. The expression patterns of *ThMYB12* were different from those of the two groups, which was down regulated at all stress time points (**Figure 4A**).

Under GA3 treatment, most of *ThMYB* genes had downregulated expression after GA3 stress in the leaves. Especially, *ThMYB2, 4, 11*, and *13* were downregulated at all stress points. In addition, the relative expression of *ThMYB6, 9* and *10* was significantly downregulated at most stress times (12–72 h). In contrast, *ThMYB3* and *ThMYB12* were mainly upregulated at most of the studied stress times. In the roots, most of the *ThMYB genes* expressed were significantly inhibited at 6 h and induced at the other studied stress time points. *ThMYB10* was significantly different than the other *ThMYBs*, which was significantly upregulated at the early stress times (6–48 h) and downregulated rapidly at 72 h after GA3 treatment (**Figure 4B**).

Under JA stress, except *ThMYB1, 9*, and *10*, almost all of the *ThMYB* genes transcribed showed downregulation at all stress points in the leaves. In addition, *ThMYB9* was highly upregulated at all of the studied stress times. The relative expression reached its peak level at 72 h (17.8-fold higher than the control). Interestingly, most of the *ThMYBs* expressed (except *ThMYB9* and *14*) showed distinct expression patterns between the leaves and roots. In the roots, except for *ThMYB1, ThMYB10* and *ThMYB14*, the other genes showed significant upregulation at all stress points. However, *ThMYB10* was significantly downregulated at all stress points (**Figure 4C**).

In general, the expression of 14 *ThMYBs* in leaf and root tissues were changed at least at one stress time point, which indicated that these *ThMYBs* might be one or several stress response genes, which might be involved in the stress tolerance of *T. hispida*. Especially, *ThMYB2, 6*, and *7* were mainly downregulated in leaves and upregulated in roots under five stress conditions. However, *ThMYB1, 3, 13*, and *14* genes were mainly upregulated in both leaves and roots in response to NaCl, ABA and PEG treatment conditions, suggesting that they might play an important role in stress tolerance. Therefore, the *ThMYB13* gene was selected for further study of stress resistance.

### Generation of Transient Expression of *ThMYB13* in *T. hispida*

**FIGURE 5:**
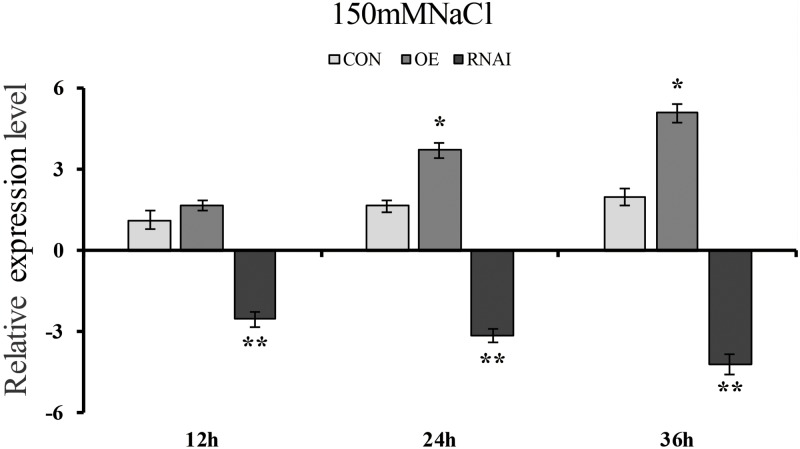
*ThMYB13* transcript levels in *T. hispida* plants with transient overexpression or knockdown of *ThMYB13*. The expression data were log2 transformed. Two-month-old *T. hispida* plants were transiently transformed with empty pROKII, 35S:MYB or pFGC:MYB. After transformation for 36 h, *T. hispida* plants were treated with 150 mM NaCl for 12, 24, or 36 h, and the expression of *ThMYB13* was determined. Con, plants transformed with empty pROKII; OE, plants transformed with 35S:MYB for overexpression of *ThMYB13*; RNAi, plants transformed with pFGC:MYB for silence the expression of *ThMYB13*. ^∗^Represents a significant difference (*P* < 0.05). ^∗∗^Represents a very significant difference (0.01 < *P* < 0.05).

To determine whether transient overexpression and suppression of the *ThMYB13* gene in *T. hispida* were successful, the expression of *ThMYB13* in Con (transformed with empty pROKII), OE and RNAi plants were investigated by qRT-PCR. The transcripts of *ThMYB13* in OE or RNAi plants was normalized by the expression of Con plants at 0 h. The results suggested that the transcript of *ThMYB13* in the OE plants was the highest among the three types of transiently transformed seedlings. At 36 h, the expression of *ThMYB13* in OE was 33.94 times that of Con. While the expression of *ThMYB13* in RNAi plants was significantly lower than those in Con, it was only 5.37% of control at 36 h (**Figure 5**). These results indicated that we successfully obtained transient overexpression and inhibition of *ThMYB13* plants.

### Physiological Characterization of Transient Expression of *ThMYB13* in *T. hispida* Under Salt Stress

**FIGURE 6:**
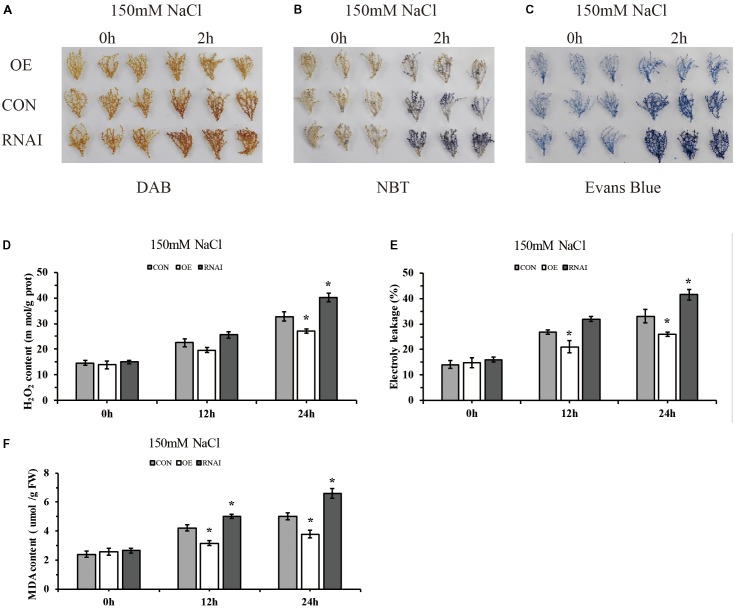
Histochemical staining and related physiological changes analyses of transformed *T. hispida*. **(A,B)** The plants were stained with DAB **(A)** and NBT **(B)** to reveal the accumulation of O^2-^ and H_2_O_2_, respectively. **(C)** Analysis of cell death by Evans blue staining. **(D)** Determination of H_2_O_2_ contents. **(E)** Analysis of cell death by measurement of electrolyte leakage. **(F)** MDA content analysis of Con, OE and RNAi plants. The experiments were conducted with three independent biological replications. ^∗^The significant difference (*t*-test, *P* < 0.05) compared with Con plants.

To preliminarily identify the function of *ThMYB13* gene, we analyzed and compared the biochemical staining and related physiological indexes of three kinds of transient transformed *T. hispida*. DAB and NBT *in situ* staining were carried out to study H_2_O_2_ and O^2-^ accumulation, respectively. In the Con, OE and RNAi plants. Oxygen ions released by H_2_O_2_ in cells can oxidize DAB to form brown precipitates, and the amount of H_2_O_2_ released from cells can be displayed according to the depth of staining. Similarly, NBT can be oxidized by superoxide ion O^2-^ to blue formazan, which can reflect the content of O^2-^ through the blue shade of formazan. The results of both NBT and DAB staining showed that the OE plants showed greatly reduced levels while RNAi plants accumulated high levels compared with Con plants (**Figures 6A,B**). The content of H_2_O_2_ was further determined. The results showed that the content of H_2_O_2_ in the three types of plants was significantly different after 24 h of salt stress, and the level of H_2_O_2_ in RNAi plants was the highest, which was 1.22 times that of Con plants. While H_2_O_2_ content in the OE plants was the lowest, it was only 82.41% of the content of Con plants (**Figure 6D**). These results indicated that in the OE plants, ROS was highly reduced, whereas in RNAi plants, ROS was accumulated highly under salt conditions. Increasing the expression of *ThMYB13* can improve ROS clearance.

To monitor the level of cell death, electrolyte leakage and evans blue staining were analyzed. Evans blue *in situ* staining indicated that in OE plants, cell death was reduced, but it was increased in that of RNAi plants when compared with Con plants exposed to salt stress (**Figure 6C**). Electrolyte leakage results confirmed these results. The relative electrical conductivities of RNAi plants were the highest at 24 h, 1.26 times that of Con plants, meanwhile those of OE were 0.78 folds those of Con plants (**Figure 6E**). At the same time, there was no difference among the three types of plants in MDA level under normal conditions. Under salt conditions, OE plants showed significantly lower MDA level than the Con plants, and the content of MDA in RNAi plants was 1.32 folds higher than MDA in the Con plants at 24 h after salt stress (**Figure 6F**). These results indicated that membrane lipid peroxidation was highly increased in RNAi plants, but was decreased in OE plants.

**FIGURE 7:**
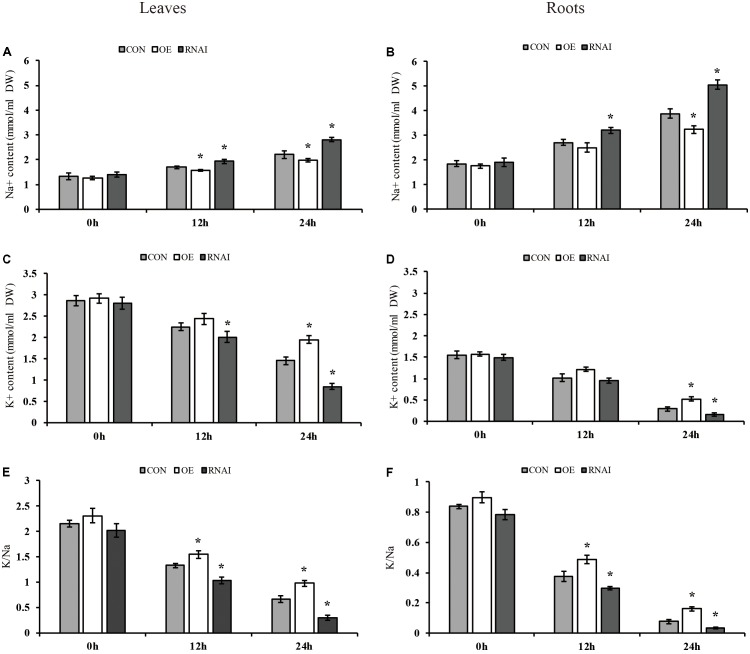
Analysis of Na^+^ and K^+^ content. Three transiently transfected plants were treated with 150 mM NaCl for 12, 24 or 36 h, in which *ThMYB13* was overexpressed at 35S: MYB (OE), the expression of *ThMYB13* was silenced with pFGC: *ThMYB13* (RNAi), or the empty pROKII plasmid was used as a control. **(A,B)** Na^+^ content in leaves **(A)** and roots **(B)**. **(C,D)** K^+^ content in leaves **(C)** and roots **(D)**. **(E,F)** K^+^/Na^+^ ratio in leaves **(E)** and roots **(F)**; ^∗^indicates significant differences between OE plants and Con, or between Con and the RNAi plants under the same conditions (*P* < 0.05).

We further determined the contents of K^+^ and Na^+^ in transformed plant roots and leaves. Under normal conditions, the sodium and potassium ion contents in the leaves and roots of three types of plants were not significantly different. Under salt conditions, all three types of plants showed higher Na^+^ content in roots than leaves. However, the RNAi plants accumulated higher Na^+^ content than the OE and Con plants, and the OE plants showed significantly lower accumulation of Na^+^ than Con plants in leaves and roots (**Figures 7A,B**). Conversely, all three types of plants showed lower K^+^ content in roots than leaves under salt stress conditions, the RNAi plants still had the lowest K^+^ content, whereas the OE plants showed the highest K^+^ contents in the leaves or roots, followed by that in the Con plants (**Figures 7C,D**). Consistently, all three types of plants displayed a higher K^+^/Na^+^ ratio in leaves than roots. At the same time, the OE plants showed the highest K^+^/Na^+^ ratio in both roots and leaves, followed by the Con plants, with the RNAi plants having the lowest K^+^/Na^+^ ratio. After salt stress for 24 h, the ratio of K^+^/Na^+^ in OE plants in leaves and roots was 1.48 and 2.13 times higher than that in Con plants, while the K^+^/Na^+^ ratio of RNAi plants was only 45.67 and 41.81% of Con plants (**Figures 7E,F**).

## Discussion

The MYB family plays an important role in plants. The MYBs function was systematically researched in Arabidopsis (Dubos et al., 2010; Mondal and Roy, 2017), *Setaria italica* (Muthamilarasan et al., 2014), *Vitis vinifera* (Wong et al., 2016), *Zea mays* (Du et al., 2012), *Populus trichocarpa* (Wilkins et al., 2009), *Gossypium raimondii* (He et al., 2016) and other plants, especially Arabidopsis and *Oryza sativa* (Katiyar et al., 2012). However, these plants are found mostly in sweet soils, and there are few studies on the ThMYB TF family and its regulatory mechanism in *T. hispida*.

In a recent study, a TaODORANT1 (R2R3-type MYB TF) from wheat, whose overexpression in tobacco was reported to enhance salt and drought tolerance by enhancing RWC and reducing H_2_O_2_, MDA, and NaCl accumulation, as well as decreasing water loss (Wei et al., 2017). Compared with the control, transgenic *BplMYB46* overexpressed birch plants increased salt and osmotic tolerance, maintained high lignin and cellulose content and reduced hemicellulose content (Guo et al., 2017). In addition, our study also confirmed the ability of the *ThMYB13* gene to increase salt tolerance through transient transformation techniques from homologous overexpression and suppression of expression, providing the basis for further investigation of the salt tolerance mechanism of *ThMYB13* gene.

In this study, 14 monomorphic and intact ORFs of *ThMYBs* were cloned. The 14 *ThMYB* genes all had the distinct characteristics of the MYB family gene and contained MYB at different positions in the protein sequence domain. In addition, the 14 ThMYB TFs were divided into 5 subgroups: class II, IV, V, VI, and X.

The MYB TFs in subgroup II were mainly involved in stress response. AT4G38620, AT4G34990, AT1G22640, AT2G16720 and AT1G35515 were involved in Arabidopsis in responding to salt stress, osmotic stress, cold acclimation, salicylic acid, ABA and JA treatments (Mondal and Roy, 2017), suggesting that *ThMYB13* TF (the member of class II MYB TFs) may also respond to abiotic stress.

The *ThMYB8* and Arabidopsis class IV members (AT4G12350, AT1G66230, AT4G22680 and AT5G16600) are closely related. In a previous study, the Arabidopsis class IV members were reported to play a role in the secondary cell wall biosynthesis regulation in Arabidopsis (Zhong et al., 2008), suggesting that *ThMYB8* may also participate in biosynthesis of secondary cell wall. The TF of subgroup VI is related to the formation of axillary meristems (Dubos et al., 2010); *ThMYB3* belongs to subgroup VI, so it is speculated that *ThMYB3* may be involved in the formation of axillary meristems.

Further, in order to analyze the abiotic stress response function, the expressions patterns of 14 *ThMYBs* in the leaves and roots of *T. hispida* in response to different abiotic stresses (salt, osmotic stress) and hormone treatments (ABA, GA3, JA) were analyzed. The expressions of most of the *ThMYBs* were significantly changed by salt and osmotic stress, ABA, GA3 and JA treatments in at least one organ. Especially, *ThMYB1, 3, 13*, and *14* genes were mainly induced in the leaves and roots of *T. hispida* under NaCl, PEG and ABA treatment conditions. *ThMYB13* was induced in the leaves and roots exposed to salt stress during the study periods. Consistently, these results further showed that *ThMYB13* might play a role in response to salt stress.

To further study the salt stress response function of *ThMYB13*, transgenic *T. hispida* plants overexpressing or knocking down *ThMYB13* and empty pROKII (control) were generated using a transient genetic transformation system. Under salt stress conditions, overexpression of *ThMYB13* displayed the lowest O^2-^, H_2_O_2_ and MDA accumulation, minimal cell death and the lowest electrolyte leakage rate among the three kinds of transiently expressed *T. hispida*. Conversely, the RNAi-silencing transiently transformed plants displayed the opposite physiological changes. In addition, our results showed that the ratio of K^+^/Na^+^ in the overexpression of *ThMYB13* was higher than that of the Con and RNAi plants when exposed to salt stress conditions. This indicated that the expression of the *ThMYB13* gene increased the uptake of K^+^ in plants and reduced the accumulation of Na^+^. It has been increasingly recognized that plant salt tolerance is directly reflected by the ratio of K^+^/Na^+^ in the cytoplasm; the higher the ratio of K^+^/Na^+^, the higher ability of salt tolerance of the plant (Ma et al., 2011; Mishra et al., 2014; Alkhateeb et al., 2015; Zang et al., 2016). These results indicated that *ThMYB13* might play an important role in tolerance to salt in transgenic *T. hispida* plants. In future studies, the salt stress regulatory mechanism of *ThMYB13* will be further studied.

## Conclusion

In this study, 14 *ThMYBs* with full ORFs were cloned and identified. The expression patterns of 14 *ThMYBs* in response to different abiotic stresses (salt and osmotic) and hormones (ABA, GA3, JA) were analyzed using qRT-PCR. The results indicated that *ThMYB13* was induced in the leaves and roots of *T. hispida* treated with NaCl at all study periods, indicating that it may involved in salt stress in plants. Further, *T. hispida* plants knockdown or overexpression of *ThMYB13* and the control transformed with empty pROKII vector were generated using a transient transformation system. Overexpression of *ThMYB13* in *T. hispida* plants displayed the lowest O^2-^, H_2_O_2_ and MDA level, minimal cell death, the most stable K^+^/Na^+^ ratio and the lowest electrolyte leakage under salt stress conditions. Conversely, the RNAi-silencing transiently transformed plants displayed the opposite physiological changes. These results suggested that *ThMYB13* might play an important physiological role in salt tolerance in transgenic *T. hispida* plants. In addition, this study may provide new insights into the function of *ThMYBs* in tolerance to abiotic stress.

## Author Contributions

TZ wrote the manuscript and performed some of the assays (data shown in **Figures 2, 5–7**). YZ and ZL performed the assays (cloned and identified the 14 *ThMYBs* with full ORFs) and analyzed the expression profiles of 14 *ThMYB* genes under different abiotic stresses and hormones by qRT-PCR; data shown in **Figures 1, 3, 4**). YW performed the data analysis and also revised the manuscript. CG provided funds for the current study, designed the study and revised the manuscript.

## Conflict of Interest Statement

The authors declare that the research was conducted in the absence of any commercial or financial relationships that could be construed as a potential conflict of interest.

## Footnotes

1 http://www.ncbi.nlm.nih.gov/orffinder/

2 http://au.expasy.org/tools/protparam.html

3 http://www.arabidopsis.org/

4 http://prosite.expasy.org/

## Supplementary Material

FIGURE S1 Multiple-sequence alignment of the 14 ThMYB proteins from *T. hispida*. Names of all the14 members were listed on the left side of the figure. Conserved amino acid residues were indicated by black shading. Conserved domains of each ThMYB protein sequence corresponds to a different position of the peptide chain.

FIGURE S2 ThMYB2, ThMYB3, ThMYB4, ThMYB8, ThMYB9 and ThMYB13 proteins conserved domain alignment. The N-terminus of its protein sequence contains two conserved MYB domains (R2, R3).

FIGURE S3 ThMYB5, ThMYB6, ThMYB7, ThMYB10 and ThMYB11 proteins conserved domain alignment. The conserved domain was present in the middle of the peptide chain.

TABLE S1 qRT-PCR primers of 14 *ThMYBs*. *α-tubulin (FJ618518), Actin (FJ618517) and β-tubulin (FJ618519)* were the internal reference genes of *T. hispida*.

TABLE S2 *ThMYB13* gene of pROKII and pFGC5941 vector primers.

## Abbreviations

ABA: abscisic acid
DAB: 3,3′-Diaminobenzidine tetrahydrochloride
GA3: Gibberellin A3
JA: jasmonic acid
MDA: malondialdehyde
Mol.Wt: molecular weight
NBT: nitroblue tetrazolium
pI: isoelectric point
qRT-PCR: real-time quantitative reverse-transcribed polymerase chain reaction
ROS: reactive oxygen species
