# Supplementary figures and images for: Comprehensive Analysis of MYB Gene Family and Their Expressions Under Abiotic Stresses and Hormone Treatments in Tamarix hispida
Source: Front Plant Sci. 2018 Sep 19;9:1303. doi: 10.3389/fpls.2018.01303 (PMC6156436; doi:10.3389/fpls.2018.01303)

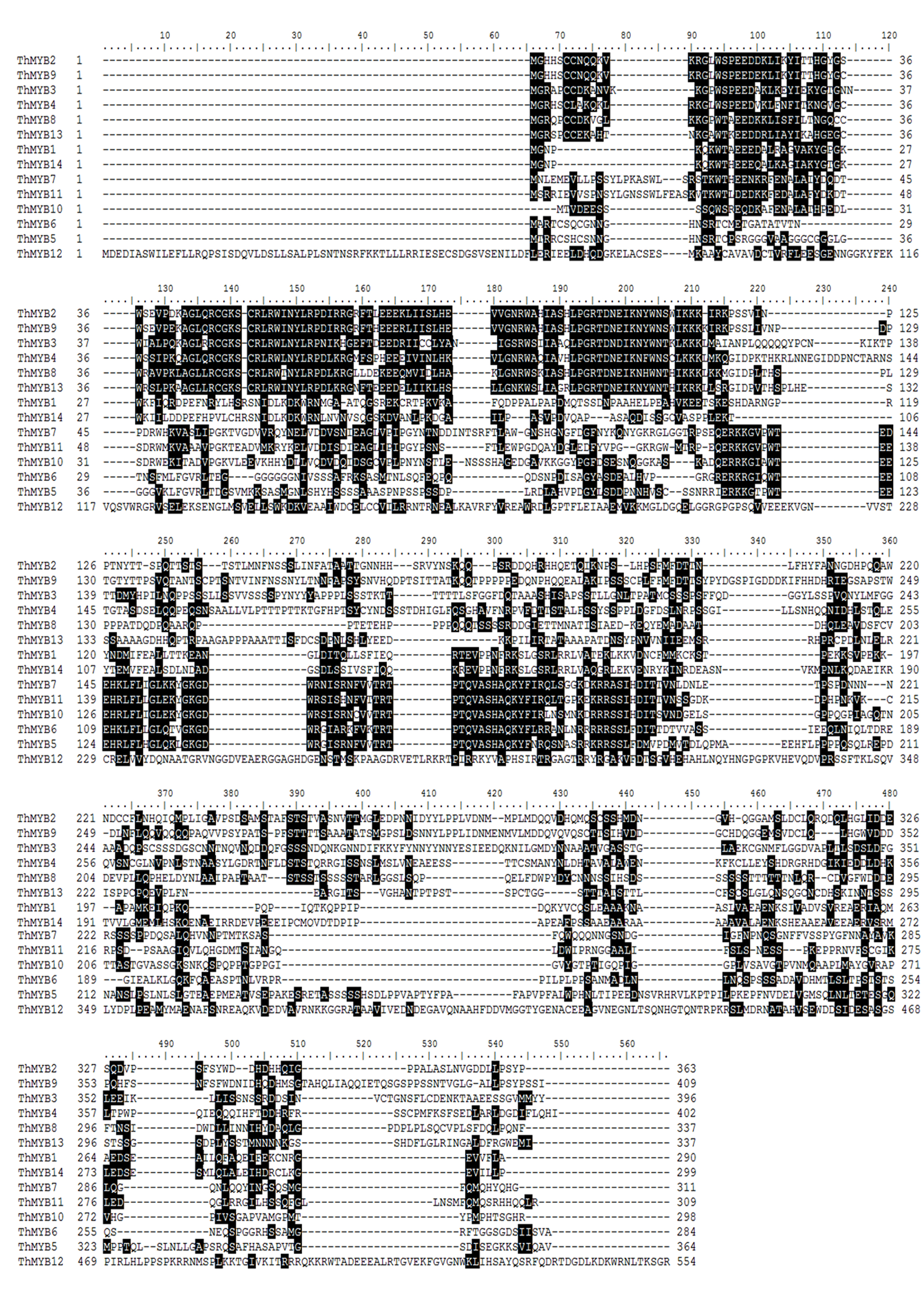

Supplement: FIGURE S1 — Multiple-sequence alignment of the 14 ThMYB proteins from T. hispida. Names of all the14 members were listed on the left side of the figure. Conserved amino acid residues were indicated by black shading. Conserved domains of each ThMYB protein sequence corresponds to a different position of the peptide chain. [file Image_1.JPEG]

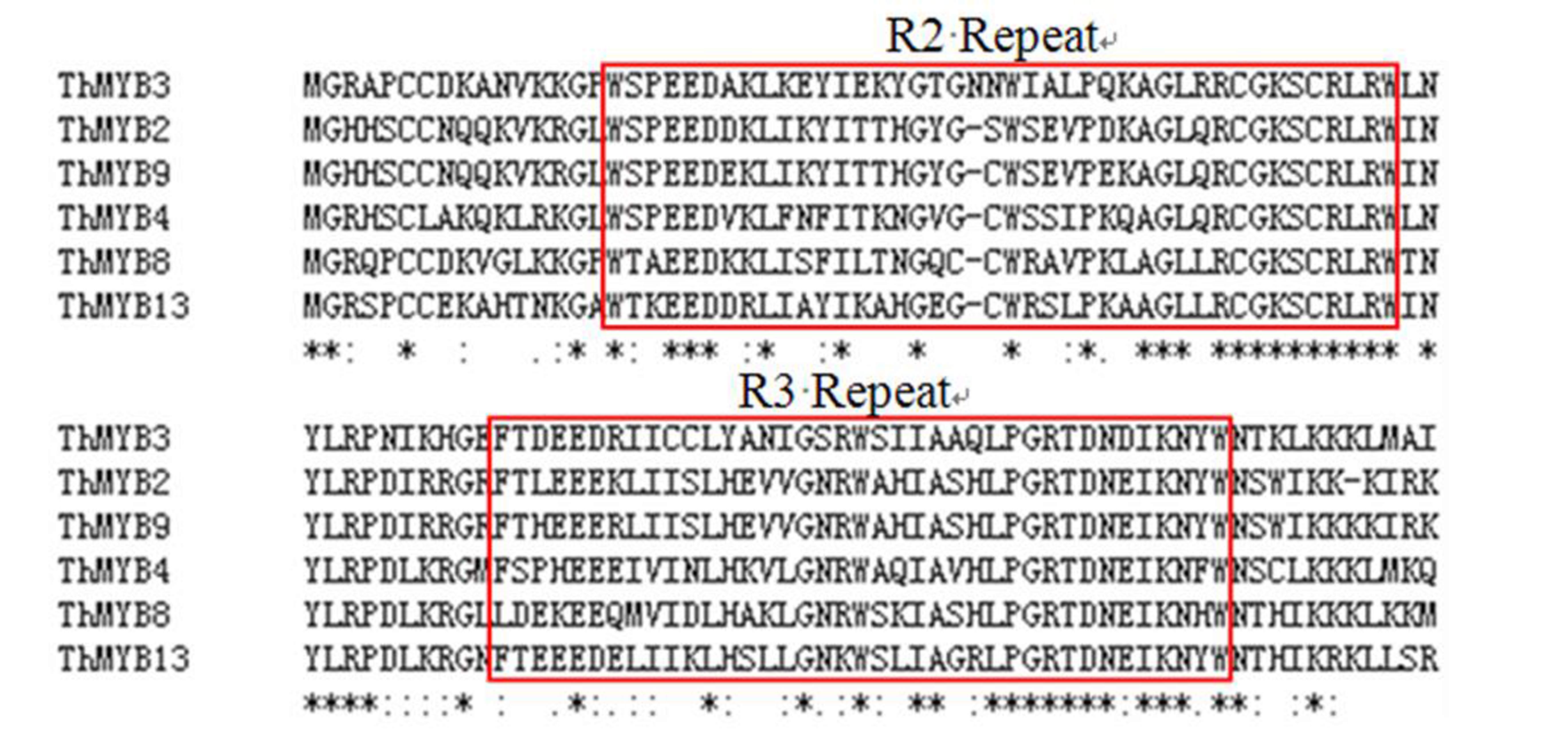

Supplement: FIGURE S2 — ThMYB2, ThMYB3, ThMYB4, ThMYB8, ThMYB9 and ThMYB13 proteins conserved domain alignment. The N-terminus of its protein sequence contains two conserved MYB domains (R2, R3). [file Image_2.JPEG]

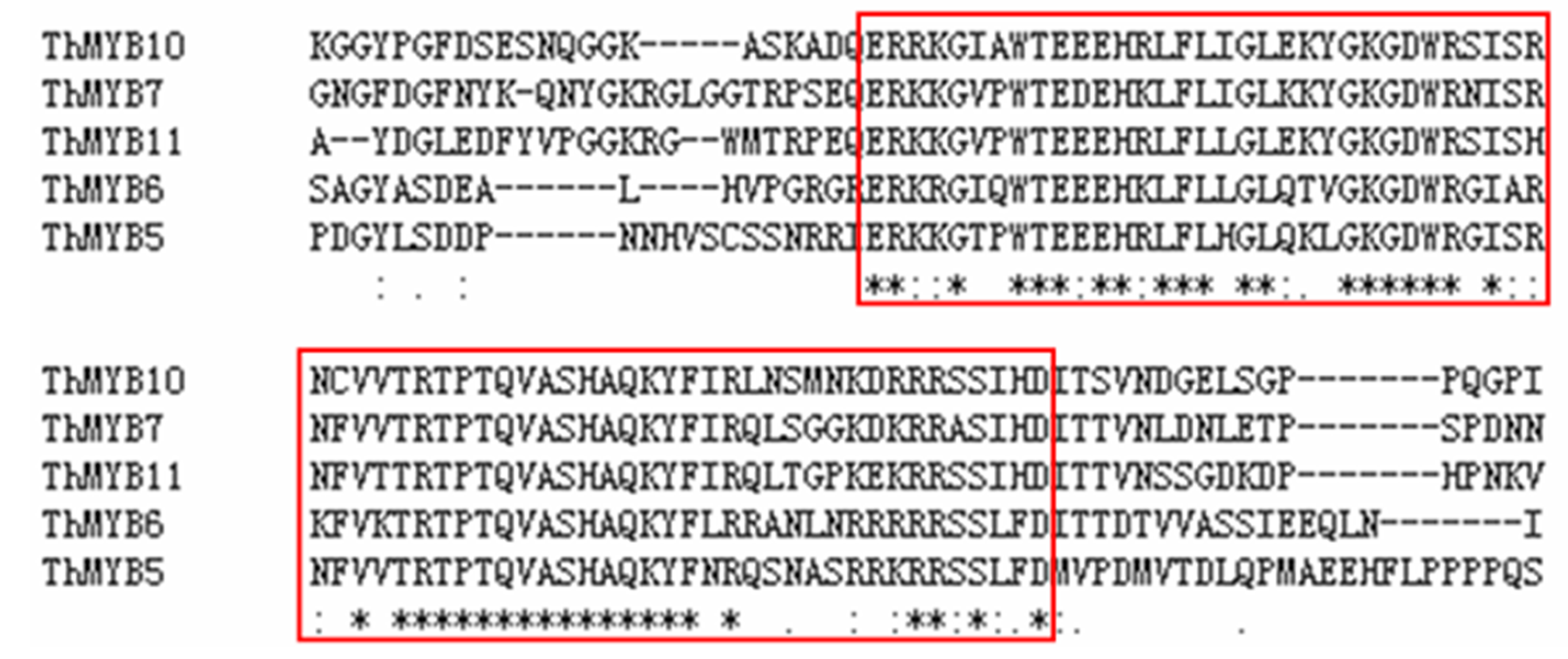

Supplement: FIGURE S3 — ThMYB5, ThMYB6, ThMYB7, ThMYB10 and ThMYB11 proteins conserved domain alignment. The conserved domain was present in the middle of the peptide chain. [file Image_3.JPEG]
