# Supplementary material for: Comprehensive Analysis of MYB Gene Family and Their Expressions Under Abiotic Stresses and Hormone Treatments in Tamarix hispida
Source: Front Plant Sci. 2018 Sep 19;9:1303. doi: 10.3389/fpls.2018.01303 (PMC6156436; doi:10.3389/fpls.2018.01303)
Supplement: TABLE S1 — qRT-PCR primers of 14 ThMYBs. α-tubulin (FJ618518), Actin (FJ618517) and β-tubulin (FJ618519) were the internal reference genes of T. hispida. [file Table_1.DOC]

Supplementary Table 1 | qRT-PCR primers of 14 *ThMYBs*. *α-tubulin (FJ618518), Actin (FJ618517) and β-tubulin (FJ618519)* were the internal reference genes of *T. hispida*.

| **Gene symbol** | **Forward Primers (5’-3’)** | **Reverse Primers (5’-3’)** |
| --- | --- | --- |
| *ThMYB1* | GGACATGCAGACATCTTCTGAT | CACCTCAGTCCTTTGCTCAAT |
| *ThMYB2* | CATGTTTGACACTACGAACCT | ACTAGAACAGGATTGCATCTG |
| *ThMYB3* | CAGCTAGTCACATTTCTGCC | GTTCTGATCATTGCTACTGC |
| *ThMYB4* | GCACGAATGCTGCAAGTTACTT | ACCGCGATCGTGACTATACTC |
| *ThMYB5* | ATGGAAGCCACTGTCAGTGAAC | ACTCGATGTCGGACACTGTTATC |
| *ThMYB6* | GGTAGTGGCTTCATCGATTG | GAGACTGCTCATTTGACTGC |
| *ThMYB7* | GAATAGTCATGGCAATGGTT | ACTTCTGTGCATGGCTAGCT |
| *ThMYB8* | GACGATGAACGCGACCATT | ACAGTAATCGTAAGGCCAGTC |
| *ThMYB9* | AGCTGTCGGCTGAGATGGATT | TGGATCAGGATTGACGATAAG |
| *ThMYB10* | GCTGGTGAAGATGGAGCAGT | TTCTGCGCATGGCTTGCTAC |
| *ThMYB11* | GGAGCATCTCGCATAACTTTG | GCGTGGTATCCAGTCAAGCT |
| *ThMYB12* | CCAGTGGAGTGCATGAACATG | ACTGCACCTTCATCATTATCT |
| *ThMYB13* | TGTCAAGACGTCATCCGCGAT | TGCTATGATCACAATTGCAAC |
| *ThMYB14* | GGATCAGATCTGAGTAGCATT | CATCTCTCCTGATCTCAGCAT |
| *Actin* | AAACAATGGCTGATGCTG | ACAATACCGTGCTCAATAGG |
| *α-tubulin* | CACCCACCGTTGTTCCAG | ACCGTCGTCATCTTCACC |
| *β-tubulin* | GGAAGCCATAGAAAGACC | CAACAAATGTGGGATGCT |
