# Supplementary material for: Comprehensive Analysis of MYB Gene Family and Their Expressions Under Abiotic Stresses and Hormone Treatments in Tamarix hispida
Source: Front Plant Sci. 2018 Sep 19;9:1303. doi: 10.3389/fpls.2018.01303 (PMC6156436; doi:10.3389/fpls.2018.01303)
Supplement: TABLE S2 — ThMYB13 gene of pROKII and pFGC5941 vector primers. [file Table_2.DOC]

*Supplementary Table 2 | ThMYB13 gene of pROKII and pFGC5941 vector primers.*

| **Gene vector name** | **Forward Primers (5’-3’)** | **Reverse Primers (5’-3’)** |
| --- | --- | --- |
| pROKⅡ-*ThMYB13* | GCTCTAGAATGGGGAGGTCTCCGTGCTGT | CGGGGTACCTCATATCATCTCCCACCCTCT |
| pFGC:*ThMYB13*-CIS | CATGCCATGGTGTCAAGACGTCATCCGCGAT | TTGGCGCGCCTGCTATGATCACAATTGCAAC |
| pFGC:*ThMYB13*-ANTI | TCCCCCGGGTGTCAAGACGTCATCCGCGAT | GCTCTAGATGCTATGATCACAATTGCAAC |
